# Identification of m^6^A-related long non-coding RNAs for predicting prognosis and immune characterizations in gastric cancer

**DOI:** 10.3389/fgene.2022.1011716

**Published:** 2022-09-26

**Authors:** Xianhui Zhang, Changjing Wang, Zhongxin Liu, Yuan Si

**Affiliations:** ^1^ Department of CT, The XingTai People’s Hospital, Xingtai, China; ^2^ Department of Gastrointestinal Surgery, The Third Hospital of Hebei Medical University, Shijiazhuang, China; ^3^ Department of Pathology, The XingTai People’s Hospital, Xingtai, China; ^4^ Endoscopic Center, The XingTai People’s Hospital, Xingtai, China

**Keywords:** gastric cancer, m^6^A, long non-coding RNA, immune, immunotherapy, prognostic model

## Abstract

**Background:** N6-methyladenosine (m^6^A) mRNA modification triggers malignant behavior in tumor cells, which promotes malignant progression and migration of gastric cancer (GC). Nevertheless, studies on the prognostic value of m^6^A-related long non-coding RNA (MRlncRNA) in GC remain quite restricted. The study aimed to develop a reasonable predictive model to explore the prognostic potential of MRlncRNAs in predicting the prognosis of GC patients and monitoring the efficacy of immunotherapy.

**Methods:** Transcriptomic and clinical data for GC were derived from TCGA. Next, univariate Cox, LASSO and multivariate Cox regression analyses were next used to identify prognostic MRlncRNAs, calculate risk scores and build risk assessment models. The predictive power of the risk models was then validated by Kaplan-Meier analysis, ROC curves, DCA, C-index, and nomogram. We attempted to effectively differentiate between groups in terms of immune cell infiltration status, ICI-related genes, immunotherapy responses, and common anti-tumor drug sensitivity.

**Results:** A risk model based on 11 MRlncRNAs was developed with an AUC of 0.850, and the sensitivity and specificity of this model in predicting survival probability is satisfactory. The Kaplan-Meier analysis revealed that the low-risk group in the model had a significantly higher survival rate, and the model was highly associated with survival status, clinical features, and clinical stage. Furthermore, the model was verified to be an independent prognostic risk factor, and the low-risk group in the model had a remarkable positive correlation with a variety of immune cell infiltrates. The expression levels of ICI-related genes differed significantly between the different groups. Lastly, immunotherapy responses and common anti-tumor drug sensitivity also differed significantly between different groups.

**Conclusion:** The risk model on the basis of 11-MRlncRNAs can serve as independent predictors of GC prognosis and may be useful in developing personalized treatment strategies for patients.

## 1 Introduction

Gastric cancer (GC) is the third leading cause of cancer-related mortality and the fifth most lethal tumor, with an incidence that widely varies across regions, i.e., >70% in developing countries, mainly in East Asia (Smyth et al., 2020; Sung et al., 2021). Just after lung cancer, GC is the second-largest malignancy in China in terms of morbidity and mortality (Chen et al., 2016). Current treatments that have been shown to be effective in gastric adenocarcinoma include systemic chemotherapy, radiotherapy, surgery, immunotherapy, and targeted therapy. However, significant therapeutic strategies are still needed for the less differentiated histologic subtypes of gastric adenocarcinoma (Sitarz et al., 2018; Joshi and Badgwell, 2021). In addition, because of genetic heterogeneity and the absence of novel treatment methods, the prognosis of patients with GC remains dissatisfactory (Yue et al., 2022). Therefore, appropriate therapies should be developed to forecast the survival probability (SP) of GC patients, better detect tumor growth, and enhance treatment results.

N6-methyladenosine (m^6^A), the most frequent modification of mRNA in eukaryotes, regulates practically all RNA cycle phases, including transcription, maturation, translation, degradation, and mRNA stability (An and Duan, 2022). The control of pathological and physiological processes, including cancer, can be influenced by m^6^A RNA methylation. Numerous research conducted in recent years has revealed that m^6^A plays a significant role in the regulation of tumors, which further controls the emergence and growth of tumors through manipulating tumor metabolism. Chen et al. (2020) reported that ALKBH5-mediated m^6^A modification of PVT1 facilitates osteosarcoma tumorigenesis, indicating that ALKBH5 and PVT1 could be potential therapeutic targets for osteosarcoma treatment. In other research, it was discovered that the TME and expressions of crucial immunological checkpoints in hepatocellular carcinoma and lung adenocarcinoma had strong connections with m^6^A-related long non-coding RNAs (MRlncRNA) profiles (Li L. et al., 2021b; Xu et al., 2021). However, further investigation of MRlncRNA signatures in GC patients is still needed.

LncRNAs refer to a class of endogenous cellular RNAs that are longer than 200bp and are encoded by the mammalian genome but are unable to create proteins due to the absence of an open reading frame (Yu et al., 2018). LncRNAs exhibit a variety of powerful capabilities in the tumor microenvironment, including tumorigenesis, tumor metastasis, and the development of associated immune diseases (Wu et al., 2020). Several combination therapies, including immunotherapy in combination with chemotherapy, surgery in combination with chemotherapy, and even drug combination therapies, have achieved significant clinical efficacy and progress (Marmarelis and Aggarwal, 2018). Therefore, it is compelling to consider combining targeted lncRNA and immunotherapy for cancer treatment.

The goal of this work was to create a MRlncRNA-based GC prognostic risk model that can predict the prognosis of the disease and the effectiveness of immunotherapy. We also hoped to gain new knowledge about the function of MRlncRNAs in GC prognosis and immunotherapy efficacy prediction.

## 2 Materials and methods

### 2.1 Data sources

The stomach cancer dataset and the matching clinical information were downloaded from TCGA (https://tcga-data.nci.nih.gov/tcga). The expression profiles of mRNAs and lncRNAs were extracted by adding annotations based on the Ensembl database (http://asia.ensembl.org). Based on previous literature and databases, we eventually acquired 23 MRGs (MRGs) (Supplementary Table S1) (Wu, 2022; Zheng et al., 2022).

### 2.2 Acquisition of MRlncRNAs and construction of risk model

In this part, MRlncRNAs were recognized applying the Pearson correlation analysis (|Pearson R| >0.4 and *p* < 0.001). The entire TCGA set was randomly assigned into a training set and a testing set (ratio, 0.7: 0.3; sample, 224: 94). The specific clinical characteristics of the training and testing sets are shown in Supplementary Table S5. There was no significant difference between the clinical characteristics of the two sets (*p* > 0.05). Next, univariate Cox analysis was employed to recognize prognostic MRlncRNAs (*p* < 0.05), LASSO analysis was applied to distinguish candidate MRlncRNAs, and a risk model was developed by utilizing multivariate Cox analysis.

### 2.3 Verification of prognostic risk model

To verify the prognostic capability of the constructed model, we calculated the risk score for each GC patient using a formula: $\overset{k}{\underset{i=1}{\sum }}\beta iSi$ , and the samples were categorized as high-or low-risk cohorts based on the median risk score. The Kaplan-Meier technique was utilized to evaluate the ability of this prognostic model to discriminate the survival differences between low-and high-risk groups. The prediction accuracy of the signature for survival in comparison to the conventional clinical characteristics and the known models was estimated by using the time-dependent receiver operating characteristic (ROC) curves and the area under the curve (AUC). On the basis of subgroups divided by clinicopathological traits, we also examined the survival disparities between various groups. Then, univariate and multivariate Cox analyses were conducted in order to verify the model as an independent predictor of prognosis. To evaluate the precision of the signature in comparison to the traditional clinical features, we also adapt decision curve analysis (DCA) and the consistency index (C-index). A nomogram combining the model and clinical features was developed to predict the 1-, 3-, and 5-year SP of patients.

### 2.4 Evaluation of the tumor immune microenvironment landscape

We further investigated the landscape of the tumor immune microenvironment and enrichment level in GC. Next, Gene Ontology (GO) and Kyoto Encyclopedia of Genes and Genomes (KEGG) enrichment analyses were carried out to evaluate the potential molecular mechanisms of the risk model. For immune infiltration calculations, we implemented the TIMER, XCELL, QUANTISEQ, MCP-COUNTER, EPIC, CIBERSORT-ABS, and CIBERSORT algorithms. This allowed us to compare immune cell subpopulations across patients with low-and high-risk. Then, investigating differences in immune function between various groups was performed by using single-sample Gene Set Enrichment Analysis (ssGSEA). Using the Wilcoxon signed rank test, the expression of immune checkpoint inhibitors (ICIs)-associated molecules in different groups was explored. The amount and quality of gene mutations among various populations were determined using a gene mutation analysis. To predict variations in immunotherapeutic responses among different groups, the tumor mutational burden (TMB) and tumor immune dysfunction and exclusion (TIDE) were calculated as well.

### 2.5 Identification of potential compounds

To evaluate therapy response and explore common anti-tumor drugs for GC treatment in the clinic, we used the R package pRRophetic to calculate the half inhibitory concentration (IC50) of drugs and make comparisons in the IC50 between different groups.

### 2.6 Cell culture

Human gastric mucosal epithelial cells GES-1 was purchased from the Beijing Institute of Cancer Research (Beijing, China) and cultured in RPMI 1640 medium containing 10% fetal bovine serum (FBS, Clark Bioscience, Claymont, United States). Human gastric adenocarcinomic AGS cell line was purchased from the National Collection of Authenticated Cell Cultures (Beijing, China) and cultured in Ham’s F-12K (Kaighn’s, Thermo Fisher Scientific, Waltham, MA, United States) medium containing 10% FBS. Human gastric cancer MKN45 cells were purchased from the Beyotime Biotechnology (Nangtong, China) and cultured in RPMI 1640 with 10% FBS. All the cells were cultured at 37°C in a 5% CO_2_ humidified incubator.

### 2.7 Quantitative real-time PCR analysis

Total RNA was extracted from GES-1, AGS and MKN45 cells using a total RNA extraction kit. Next, 1 μg of total RNA was reverse transcribed into cDNA with the iScript cDNA synthesis kit. A Bio-Rad CFX96 system was used to perform quantitative real-time PCR (qPCR) analysis, and the relative mRNA levels were calculated using the 2−ΔΔCt method using GAPDH for normalization. The primer sequences to amplify the genes encoding LASTR, AC008808.1, AC027601.5, AC025766.1, LINC00454 and AL139147.1, and GAPDH are listed in Supplementary Table S10.

## 3 Results

### 3.1 Identification of NRlncRNAs

**FIGURE 1:**
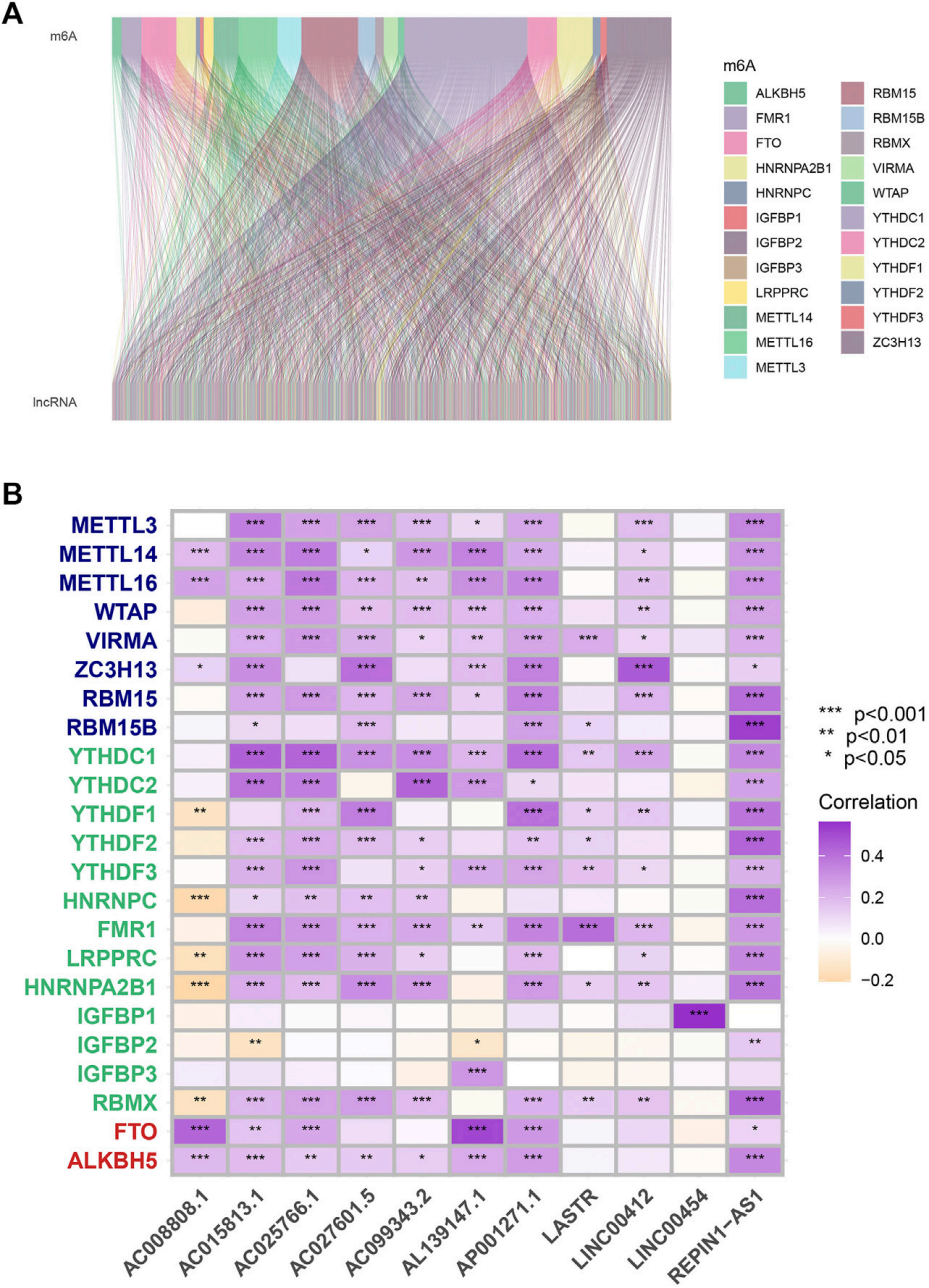
Process of MRlncRNAs identification. **(A)** Sankey relational diagram for all MRlncRNAs and the 23 MRGs. **(B)** The correlations between 23 MRGs and the 11 prognostic MRlncRNAs.

According to co-expression analysis, 979 MRlncRNAs were recognized (cor > 0.4 and *p* < 0.001) (Supplementary Table S2). Finally, the m^6^A-lncRNA co-expression network was visualized by using the Sankey diagram in Figure 1A. 11 lncRNAs were selected for the prognostic risk model and, the correlation between MRGs and MRlncRNAs is presented in Figure 1B.

### 3.2 Construction and verification of the risk model

**FIGURE 2:**
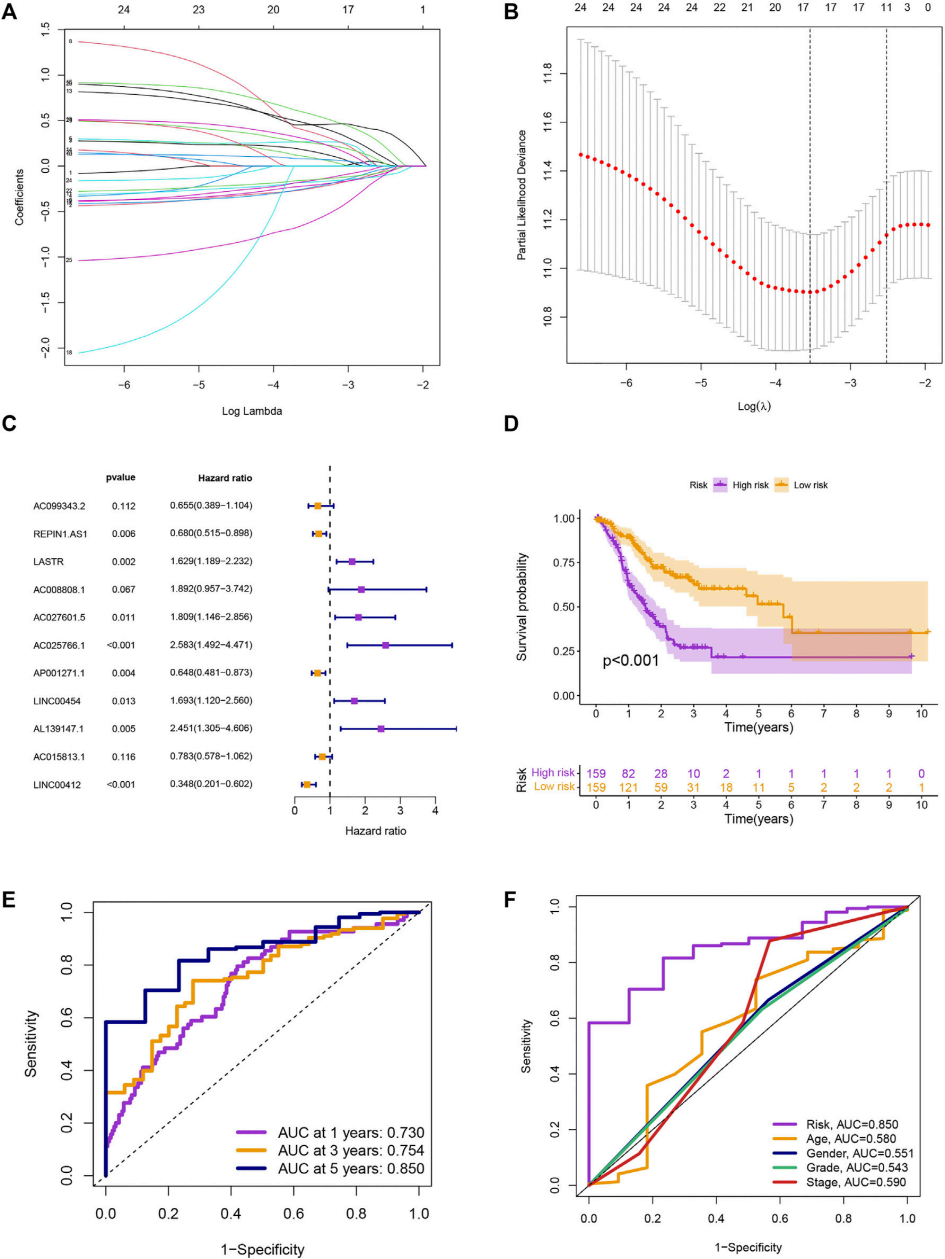
Development of risk model. **(A)** The LASSO coefficient profile. **(B)** Coefficient profile plot. **(C)** Multivariate Cox regression analysis showed 12 prognostic lncRNAs. **(D)** Kaplan-Meier curves of the SP in different risk groups. **(E)** ROC curves to predict the sensitivity of 1-, 3-, and 5-year survival. **(F)** ROC curves to predict the sensitivity of the risk grade and other clinicopathological characteristics.

We screened 25 prognostic MRlncRNAs from 979 MRlncRNAs through univariate Cox regression analysis (Supplementary Figure S1A and Supplementary Table S3). Out of 25 prognostic MRlncRNAs, 17 potential MRlncRNAs were chosen by applying the LASSO analysis (Figures 2A,B). Finally, the multivariate Cox analysis was used to create a prognostic risk model that included 11 MRlncRNAs (Figure 2C and Supplementary Table S4). Based on median risk scores, GC patients were categorized into low-risk and high-risk groups. The survival analysis of these two groups suggested that the SP of the low-risk group was higher (*p* < 0.001) (Figure 2D). The 1-year, 3-year, and 5-year survival rates of GC patients are predicted by adopting this risk model, and the prediction accuracy rates are displayed in Figure 2E. The model also demonstrated a higher AUC than other clinicopathological characteristics such as age, gender, grade, and stage, indicating that it was comparably reliable (Figure 2F).

### 3.3 Assessment of the risk assessment model and clinical characteristics

**FIGURE 3:**
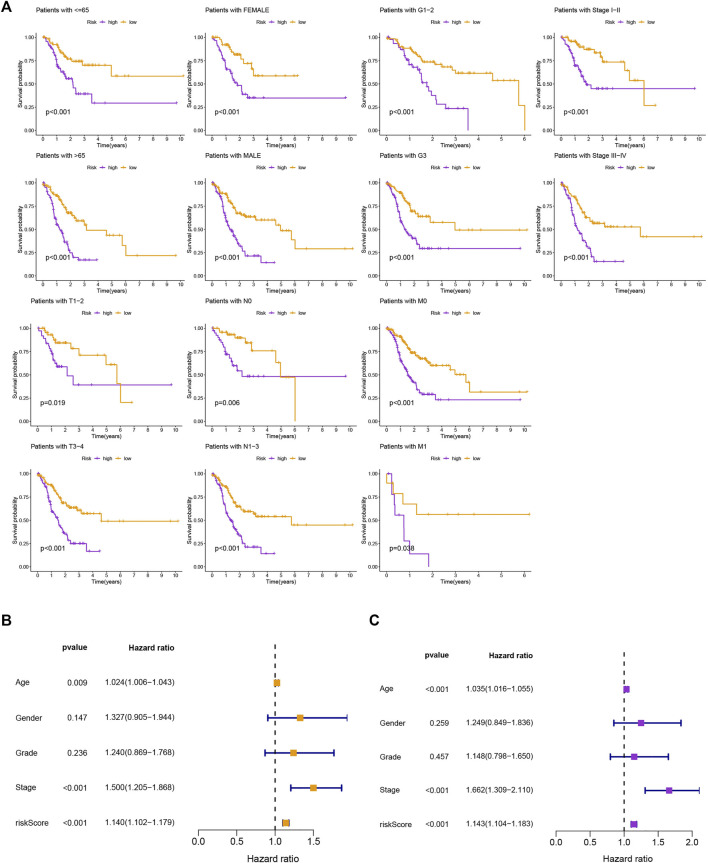
Kaplan-Meier curves of SP and PCA between two groups. **(A)** Kaplan-Meier curves of SP differences stratified by age, gender, clinical stage, or T stage between different risk groups. **(B,C)** Univariate and multifactorial Cox analyses showed that risk score is a risk factor for individual prognosis.

The survival prediction and ROC curves of the testing and entire sets were shown in Supplementary Figures S1B–E, indicating the prediction accuracy of this risk model is satisfactory. In the subgroups separated by age (≤65 or >65), gender (female or male), clinical stage (G1-2 or G3, stage Ⅰ-Ⅱ or stage Ⅲ-Ⅳ), or TNM stage (T1-2 or T3-4, N0 or N1-3, M0 or M1), the SP was higher in the low-risk group, which indicated that the constructed model was appropriate for various circumstances (Figure 3A). Univariate and multivariate Cox analyses were implemented to evaluate whether this 11 MRlncRNAs risk model had independent prognostic features for GC patients. The hazard ratio of the risk score and the 95% confidence interval were 1.140 and 1.102–1.179 (*p* < 0.001) in univariate Cox regression analysis, respectively (Figure 3B). According to multivariate Cox regression analysis, the hazard ratio was 1.143 and the 95% confidence interval was 1.104–1.183 (*p* < 0.001) (Figure 3C), indicating that the risk model of the 11 MRlncRNAs was independent and had nothing to do with clinicopathological features including age, gender, clinical stage, T stage, and risk scores (Supplementary Table S6).

**FIGURE 4:**
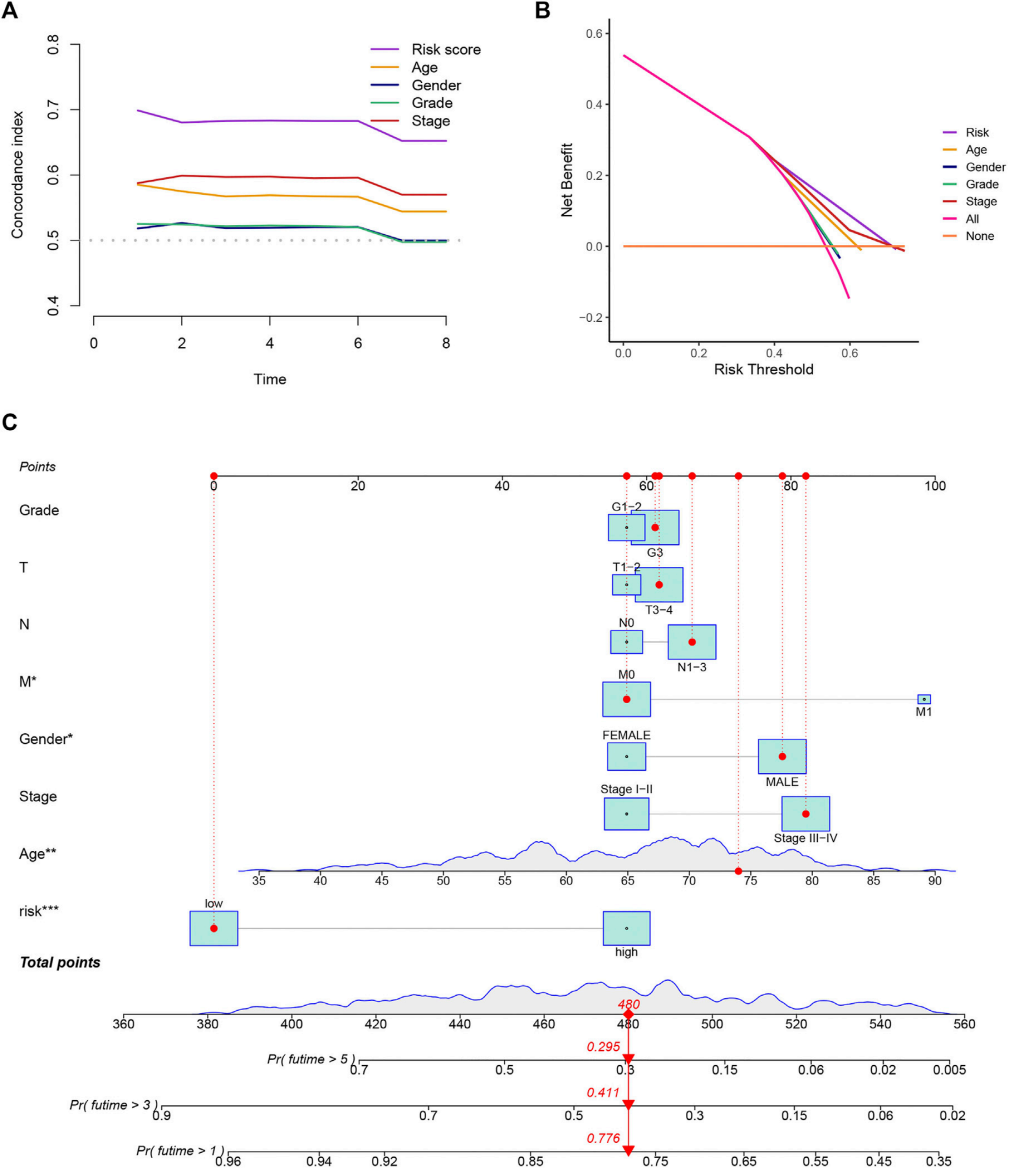
Construction of a nomogram. **(A,B)** The results of the C-index and DCA indicated that the model better predicted the prognosis of GC than other traditional clinical characteristics. **(C)** The nomogram was constructed according to the risk assessment model and clinical features.

To evaluate the precision of the signature in comparison to the traditional clinical features, we also adopted the C-index and DCA, demonstrating that the signature has a greater ability to forecast the prognosis of GC than other clinical features (Figures 4A,B). We combined this risk model with clinicopathologic characteristics and evaluated patients’ total risk scores according to them to build a nomogram to predict 1-year, 3-year, and 5-year survival rates of GC patients (Figure 4C).

### 3.4 Estimation of the immune landscape

**FIGURE 5:**
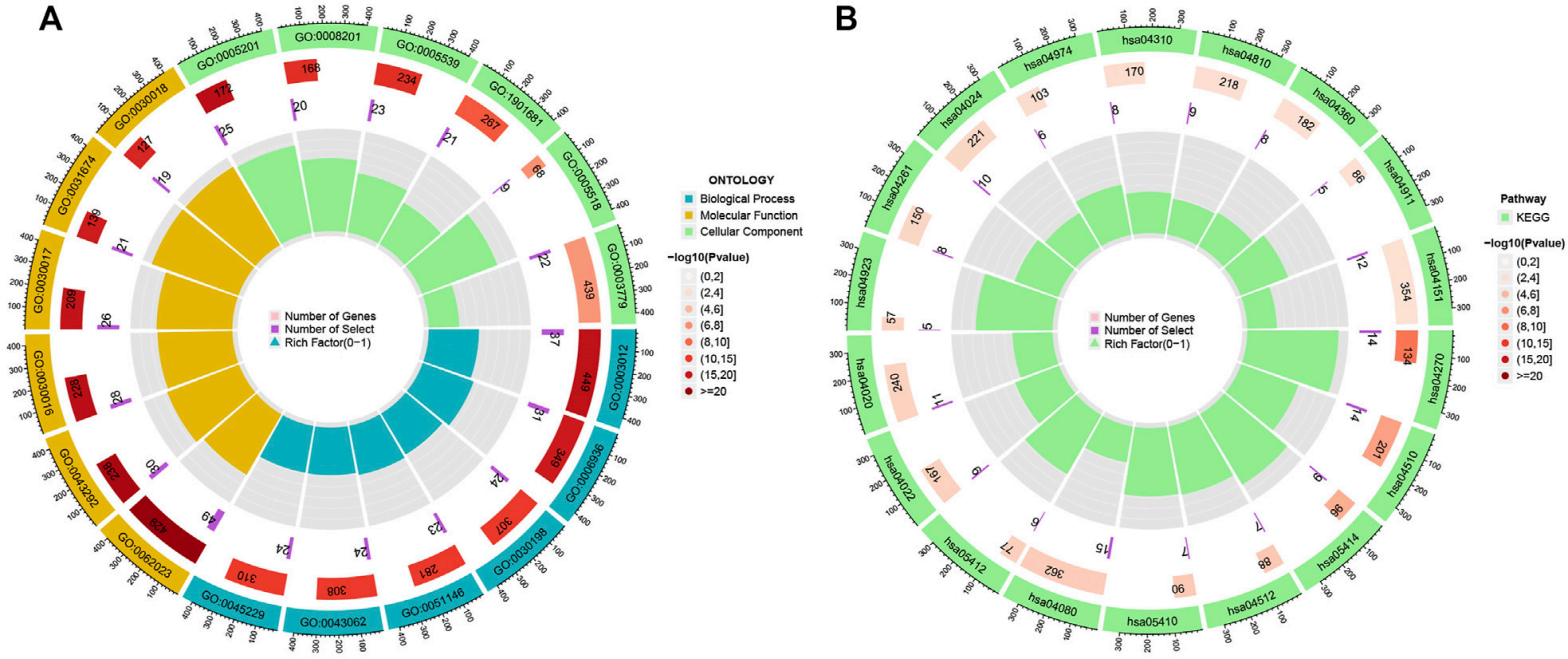
Potential functional and pathways in the model. **(A)** GO enrichment analysis. **(B)** KEGG enrichment analysis.

**FIGURE 6:**
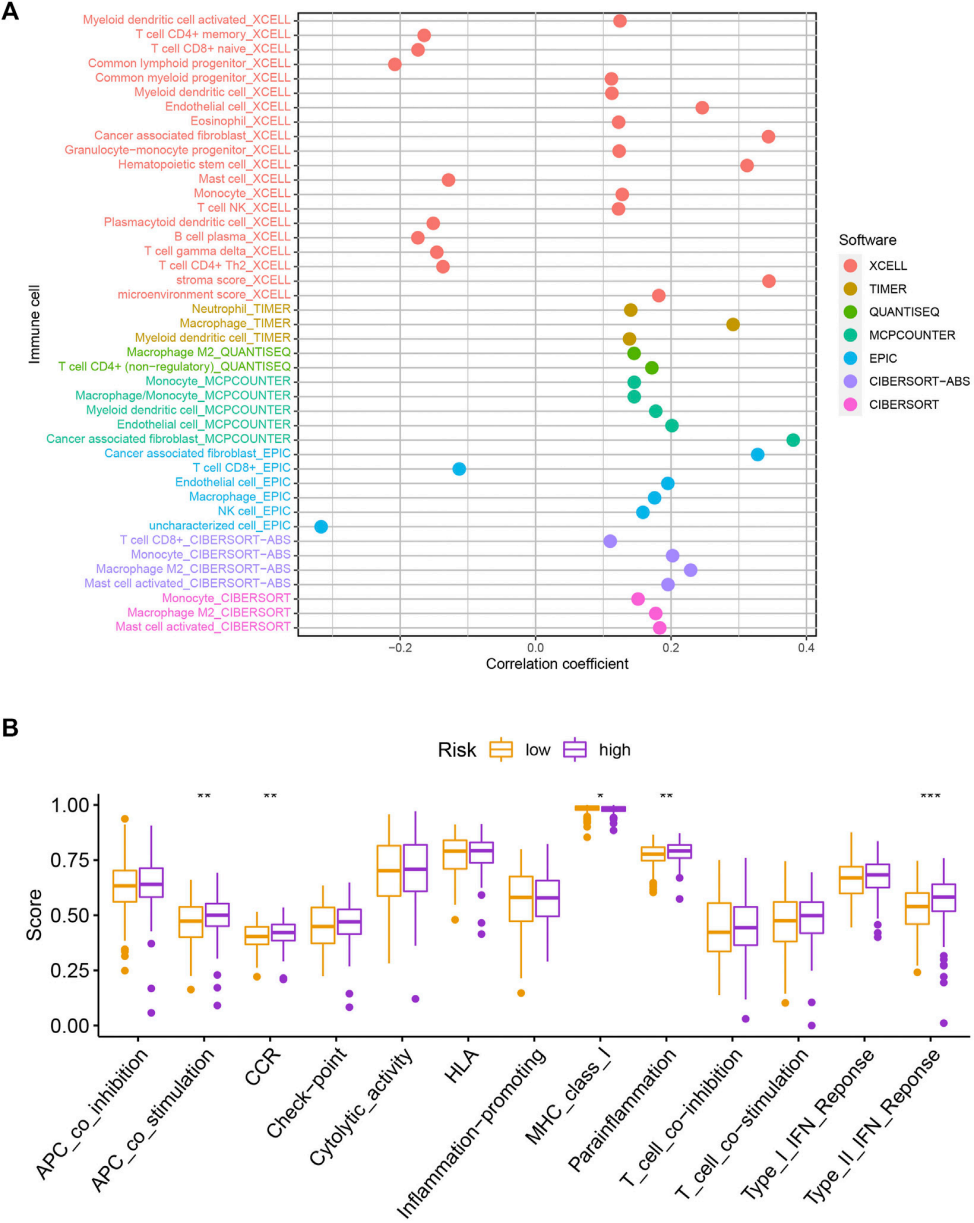
Assessment of the immune landscape. **(A)** Changes in immune cell infiltration that are identified by the MRlncRNA-based risk model are linked to an increased chance of developing GC. **(B)** Several immune functions were statistically different between the two groups.

To explore the potential functional and pathway differences in different risk groups, we identified 347 differentially expressed genes in high-and low-risk groups for GO and KEGG enrichment analysis. The results of GO and KEGG enrichment analysis are presented in Figures 5A,B, and their details are shown in the Supplementary Tables S7, S8. By examining potential connections between immune cell sub-populations and GC risk, we explored whether the prognostic MRlncRNA pairings included in the risk model were connected to activities in the tumor immune milieu. We found a statistically significant association between changes in the immune cell landscape and elevated GC risk (Figure 6A and Supplementary Table S9). Statistical differences were found in some immune functions, including parainflammation, response to type II interferon, CCR, and APC stimulation (Figure 6B).

### 3.5 Evaluation of cancer immunotherapy response and drug sensitivity

**FIGURE 7:**
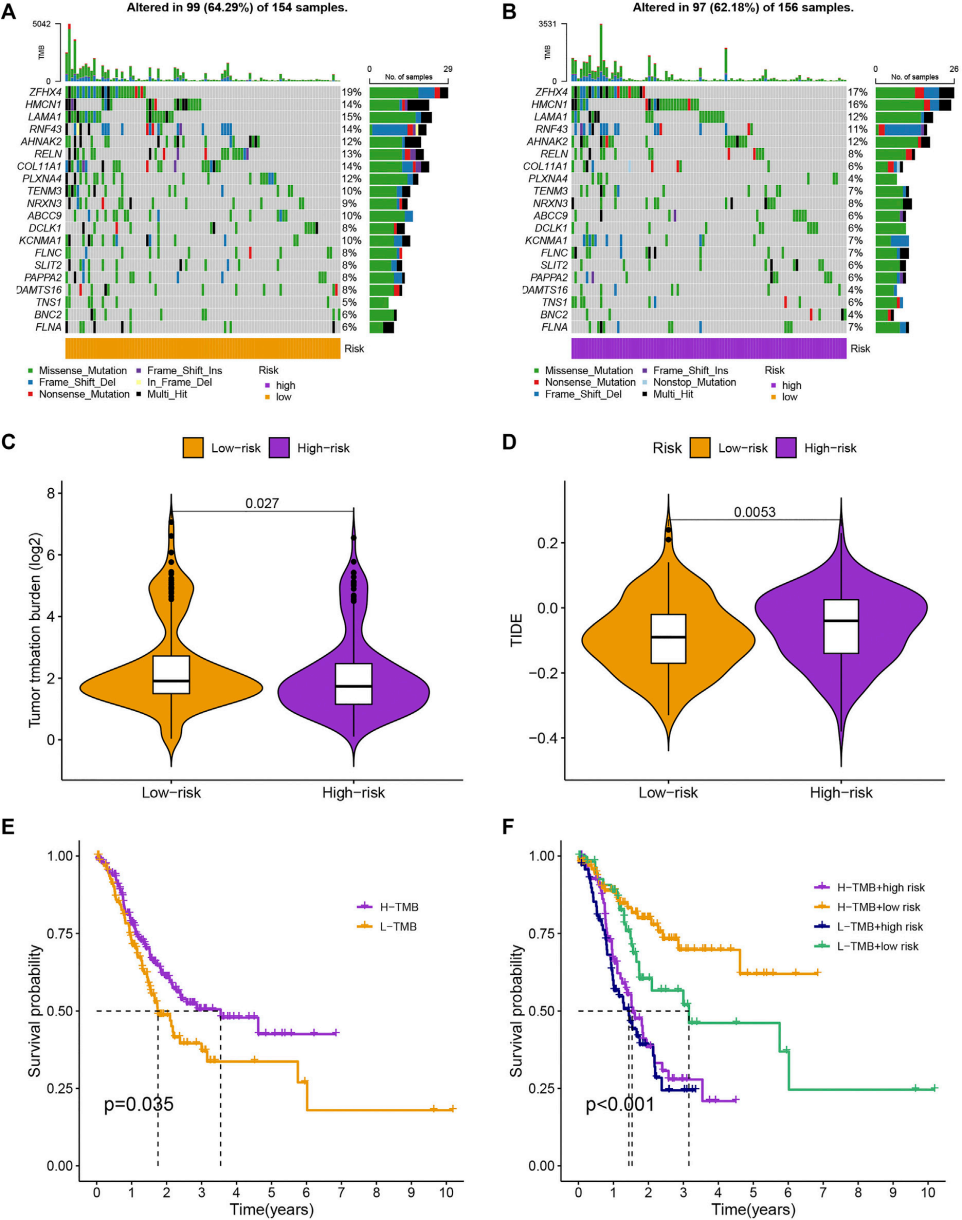
Evaluation of cancer immunotherapy response. **(A,B)** More genes were mutated in the high-risk group. **(C,D)** Comparation of the immunotherapy response of the different-risk group to predict TIDE and TMB. **(E,F)** The SP of patients in different subgroups.

**FIGURE 8:**
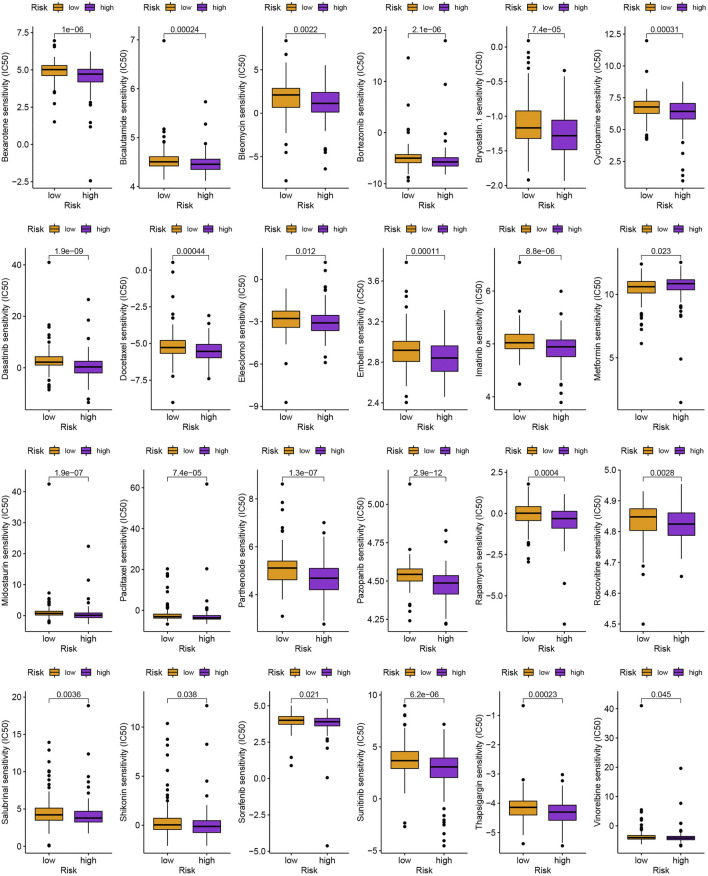
Identification of commonly used anti-tumor drugs targeting the model.

As is shown in Figures 7A,B, ZFHX4, HMCN1, LAMA1, RNF43, AHNAK2, RELN, COL11A1, PLXNA4, TENM3, and NRXN3 were the top 10 most mutated genes for differentially expressed genes between high-and low-risk groups. ZFHX4, LAMA1, and RNF43 mutations were much more high in patients in the low-risk group than in those in the high-risk group, but the exact reverse was seen for the HMCN1 mutation levels. The relationship between the MRlncRNAs model and immunotherapeutic biomarkers was then examined. As expected, we observed a significantly greater response to immunotherapy in the high-risk group than in the low-risk group, indicating that our m^6^A-based classifier score can be applied for TIDE and TMB prediction (Figures 7C,D). Next, based on TMB, we categorized all samples in the high-and low-risk groups into two subgroups: high-TMB and low-TMB, respectively. The patients with low TMB after immunotherapy had a higher SP (Figure 7E). Then, we predicted the SP of patients with different TMB subgroups in the two risk groups separately and found that patients with low TMB in the low-risk group had the highest probability of survival (Figure 7F). This implies that we can select appropriate immunotherapeutic agents for GC patients according to their risk patterns. In drug susceptibility analysis, statistical differences were found in the IC50 among the 24 chemical or targeted drugs used for GC treatment in the different groups (Figure 8).

### 3.6 Quantitative real-time PCR analysis

**FIGURE 9:**
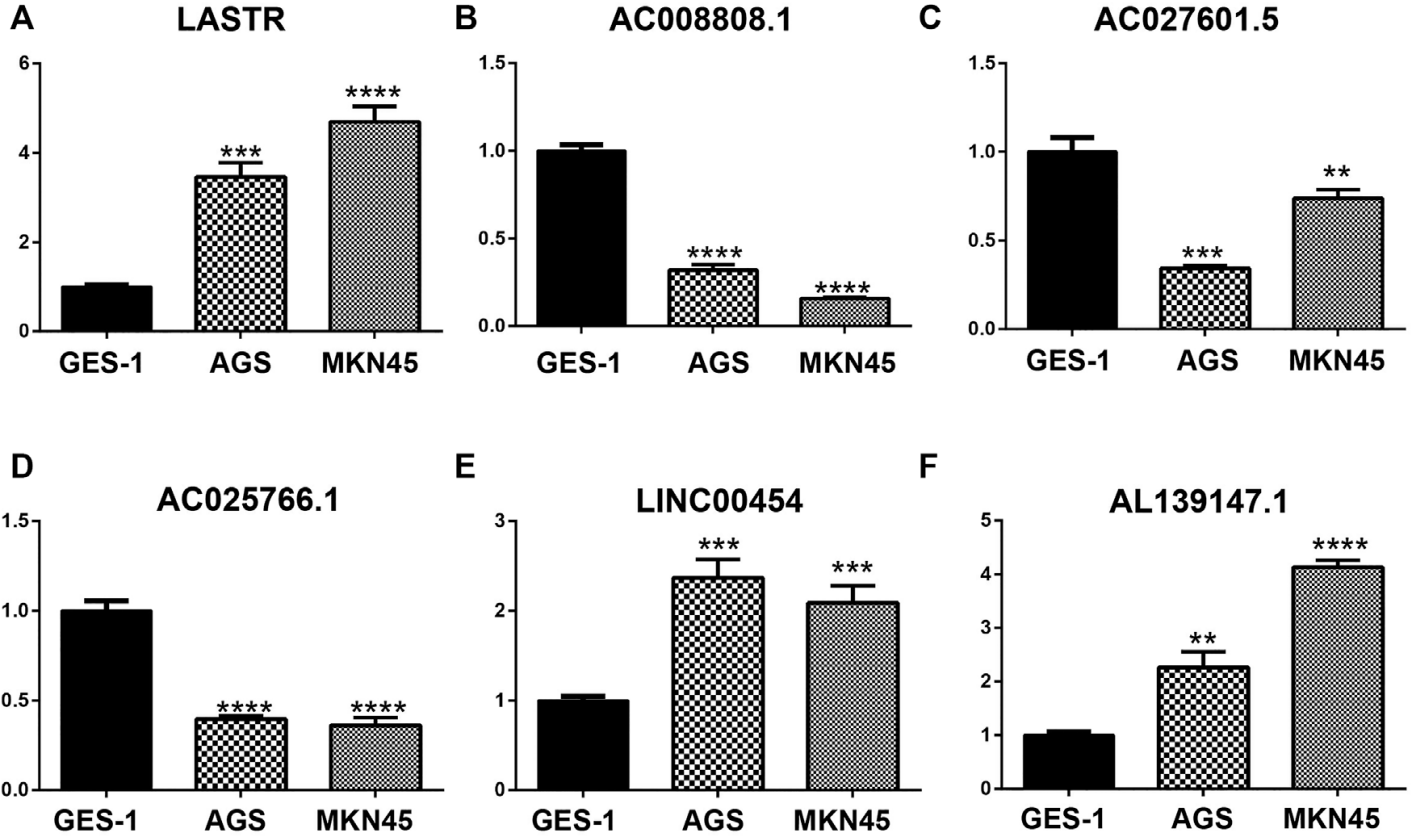
The mRNA levels of LASTR, LINC00454, and AL139147.1 were increased in AGS and MKN45 cells, whereas the mRNA levels of AC008808.1, AC027601.5, and AC025766.1 were decreased in AGS and MKN45 cells.

The qPCR results showed that the mRNA levels of LASTR, LINC00454 and AL139147.1 were increased in AGS and MKN45 cells, whereas the mRNA levels of AC008808.1, AC027601.5 and AC025766.1 were decreased in AGS and MKN45 cells, which further validates the prognostic risk model we have constructed (Figure 9).

## 4 Discussion

GC is the third most common cause of cancer-related death and the fifth most deadly malignancy, and the prognosis of GC patients remains unsatisfactory (Sexton et al., 2020). It is crucial to create innovative methods to raise the survival rate of this illness due to the dismal prognosis of patients with late-stage GC (Johnston and Beckman, 2019). Immunotherapy is currently regarded as a cutting-edge treatment option for diseases like breast, stomach, and lung cancer. In addition, lncRNAs have been demonstrated to have a significant role in the regulation of gene expression in various malignancies, including GC (Denaro et al., 2019). Therefore, we created a prognostic model based on MRlncRNAs in GC to predict the prognosis of GC patients, explore TME and cancer immunotherapy responses, and add fresh ideas to the clinical treatment of GC.

In our study, we identified 979 MRlncRNAs from the TCGA to discuss the prognostic function of MRlncRNAs. We identified 000 prognostic MRlncRNAs and a risk model to predict SP in GC patients was built based on 11 MRlncRNAs: AC099343.2, REPIN1. AS1, LASTR, AC008808.1, AC027601.5, AC025766.1, AP001271.1, LINC00454, AL139147.1, AC015813.1, and LINC00412. Of these, AC099343.2 was identified as an autophagy-related lncRNA signature for potential prognostic biomarkers of patients with cervical cancer (Feng et al., 2021); REPIN1. AS1 and LASTR were developed to improve the prognosis prediction of stomach adenocarcinoma patients (Luo et al., 2022). In another study, AP001271.1 and three other lncRNAs were selected to construct a risk model for predicting prognosis for GC patients (Wei et al., 2021). Furthermore, other lncRNAs were found for the first time in this study.

Then, patients were grouped into low- and high-risk groups according to median risk score, and some analyses were made, including Kaplan-Meier analysis, univariate and multivariate Cox analyses. We found that the MRlncRNAs risk model was an independent risk factor of SP. Through ROC analysis, we also discovered that the model was more accurate than conventional clinical features in predicting GC survival. Finally, according to a nomogram developed to make predictions in SP of GC patients, we found that the predicted and measured values for the SPs are highly consistent. On the basis of the above analysis, this risk model based on 11 MRlncRNAs that were independently related to SP was pretty accurate.

TMB, which stands for total number of somatic coding mutations, has gained much attention as a new predictive biomarker that is closely related to the development of neoantigens that trigger anti-tumor response (Allgäuer et al., 2018; Addeo et al., 2021). The context of TMB identified at diagnosis represents the immune response and chemotherapy benefit, and variations in the numbers of CD8^+^ T cells, CD4^+^ T cells, macrophages, and cancer-associated fibroblasts infiltrating in the TME correlate with clinical outcomes in a variety of malignancies, including GC, melanoma, urothelial cancer, lung cancer, and breast cancer (Zeng et al., 2019; DeBerardinis, 2020). In our study, we discovered that the TMB of the low-risk group was higher than that of the high-risk group. Furthermore, the TIDE prediction score has also been deployed in numerous investigations, and its ability to predict prognosis has been successfully established (Jiang P. et al., 2018a; Chen et al., 2022). In our study, the TIDE algorithm predicted a more favourable response to immunotherapy in individuals with high-risk subtypes. Based on the aforementioned results, we draw the conclusion that our prediction model could deliver precise immunological biomarkers for oncology. What’s more, the conclusions of our study provide information on the molecular biology of lncRNAs that are connected to m^6^A in GC. In this study, the TIDE algorithm predicted that immunotherapy is more effective for patients with the high-risk subtype. We come to the conclusion that our prediction model could provide accurate immunological indicators for oncotherapy based on the aforementioned findings. Additionally, the molecular biological mechanisms of MRlncRNAs in GC are also newly revealed by this work.

In general, TNM stage is the most important determinant of GC prognosis in clinical practice (Jiang Y. et al., 2018b). However, due to tumor heterogeneity, even patients with similar TNM staging exhibit widely varying prognoses (Li K. et al., 2021a). It suggests that extant periodization algorithms are deficient in capturing GC heterogeneity and accurately predicting prognosis in GC patients. Therefore, more research remains to be carried out in investigating potential predictive and therapeutic biomarkers. The study suggests that MRlncRNA models may provide a new tool for GC prognosis prediction. This study used several analytical methods to validate this new model so that we could select the optimal model and apply it in a rational way. We hypothesized that the predictive model would still be feasible without external data validation. However, we are aware that there are flaws and limitations in this study. As the molecular mechanism of MRlncRNAs is not fully understood, it would be sensible to validate this with more convincing basic experiments. And the sample size needs to be expanded in future studies to increase the confidence. Furthermore, we will investigate the role of MRlncRNAs and their interactions with MRGs through *in vitro* experiments, seeking to assess the accuracy of the model in future studies and provide new ideas for clinical treatment.

## 5 Conclusion

In summary, this study reveals that the processes and mechanisms of MRlncRNAs are based on a novel prognostic model that provides new insights into GC prognosis prediction and clinical treatment. Furthermore, the model we developed was accurate and effective in predicting GC prognosis and showed sensitivity in identifying GC patients who responded well to immunotherapy.

## Data Availability

The original contributions presented in the study are included in the article/Supplementary Material; further inquiries can be directed to the corresponding author.

## Supplementary material

Supplementary Figure S1
**(A)** 25 prognostic MRlncRNAs recognized by univariate Cox analysis. **(B–E)** Kaplan-Meier survival curves, and 1, 3 and 5-year ROC curves in the testing set and total set.

Supplementary Table S1 23 MRGs.

Supplementary Table S2 The results of MRGs-MRlncRNAs.

Supplementary Table S3 25 prognostic MRlncRNAs were selected through univariate Cox regression analysis.

Supplementary Table S4 11 MRlncRNAs were selected by the multivariate Cox analysis.

Supplementary Table S5 There was no significant difference between the clinical characteristics of the two sets.

Supplementary Table S6 The details of univariate and multivariate Cox analyses.

Supplementary Table S7 The details of GO enrichment analysis.

Supplementary Table S8 The details of KEGG enrichment analysis.

Supplementary Table S9 The details of immune cell landscape.

Supplementary Table S10 Primer sequences for quantitative real-time PCR analysis.
